# Chromosome-level genome assembly and annotation of Zicaitai (*Brassica rapa var. purpuraria*)

**DOI:** 10.1038/s41597-023-02668-0

**Published:** 2023-11-03

**Authors:** Hailong Ren, Donglin Xu, Wanyu Xiao, Xianyu Zhou, Guangguang Li, Jiwen Zou, Hua Zhang, Zhibin Zhang, Jing Zhang, Yansong Zheng

**Affiliations:** 1 Guangzhou Academy of Agricultural Sciences, Guangzhou, 510308 China; 2 Crops Research Institute, Guangdong Academy of Agricultural Sciences/Guangdong Provincial Key Laboratory of Crop Genetic Improvement, Guangzhou, 510640 China; 3 National Key Laboratory of Cotton Bio-breeding and Integrated Utilization, Institute of Cotton Research, Chinese Academy of Agricultural Sciences, Anyang, 455000 China; 4 Zhengzhou Research Base, National Key Laboratory of Cotton Bio-breeding and Integrated Utilization, Zhengzhou University, Zhengzhou, 450000 China

**Keywords:** Genome informatics, Genome

## Abstract

Zicaitai is a seasonal vegetable known for its high anthocyanin content in both stalks and leaves, yet its reference genome has not been published to date. Here, we generated the first chromosome-level genome assembly of Zicaitai using a combination of PacBio long-reads, Illumina short-reads, and Hi-C sequencing techniques. The final genome length is 474.12 Mb with a scaffold N50 length of 43.82 Mb, a BUSCO score of 99.30% and the LAI score of 10.14. Repetitive elements accounted for 60.89% (288.72 Mb) of the genome, and Hi-C data enabled the allocation of 430.87 Mb of genome sequences to ten pseudochromosomes. A total of 42,051 protein-coding genes were successfully predicted using multiple methods, of which 99.74% were functionally annotated. Notably, comparing the genome of Zicaitai with seven other species in the *Cruciferae* family revealed strong conservation in terms of gene numbers and structures. Overall, the high-quality genome assembly provides a critical resource for studying the genetic basis of important agronomic traits in Zicaitai.

## Background and Summary

**Fig. 1:**
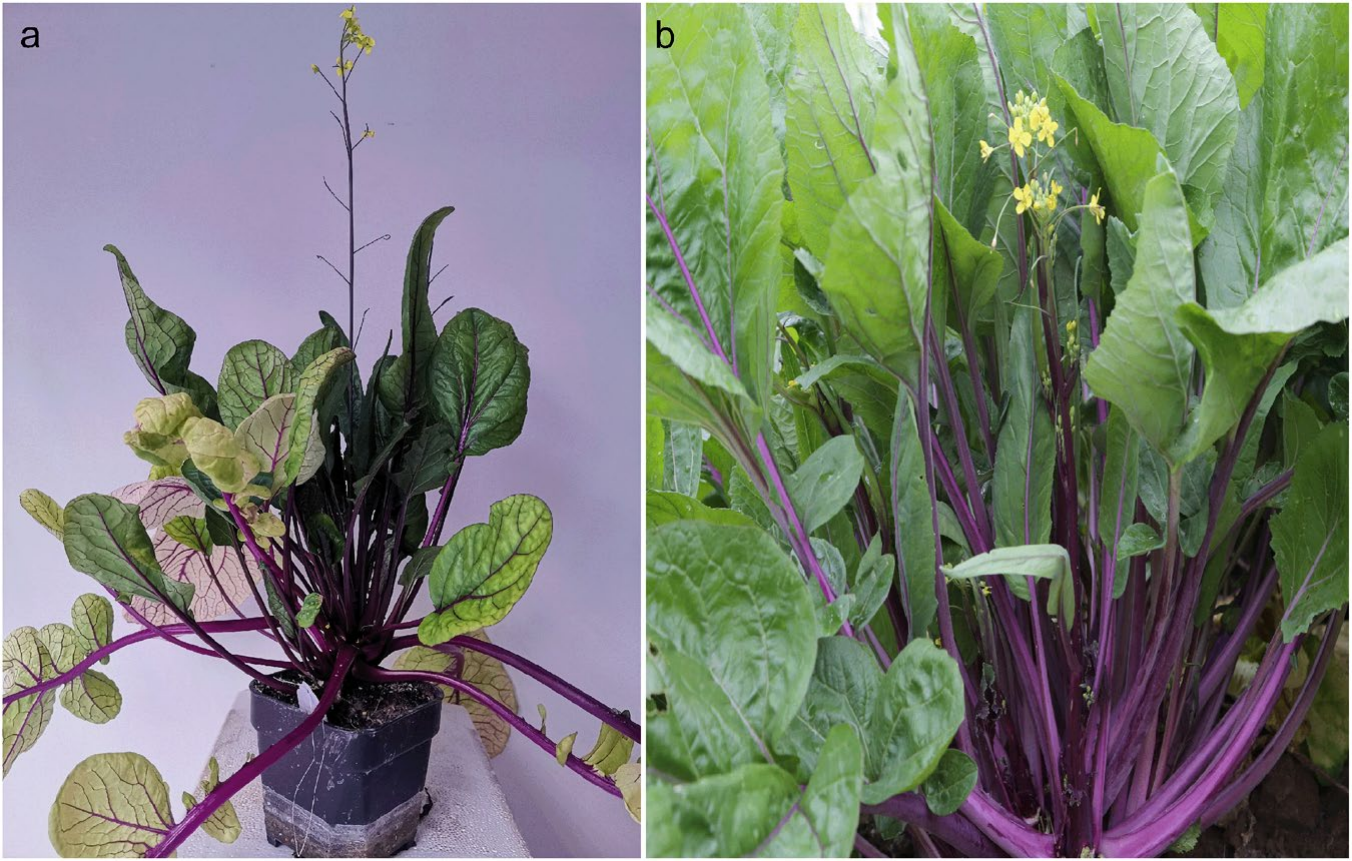
Images of Zicaitai mature plant in laboratory research (**a**) and field experiment (**b**).

*Brassica rapa* var. *purpuraria* (NCBI: txid386281, Fig. 1) belongs to the *Cruciferae* family^1,2^ and is named “Zicaitai” for its purple stalks^3^. Zicaitai originated in the southern regions of China and then spread to the Yangtze River Basin, where it was subsequently widely domesticated. Its cultivation history can be traced back to ancient China, spanning more than a thousand years. To date, Zicaitai is a popular vegetable in China, and is also exported to other countries in Asia, Europe, and America.

The stems and leaves of Zicaitai are the main edible portion and are rich in anthocyanin^4–6^. Anthocyanin is a water-soluble natural pigments, that possesses anti-cancer, anti-viral, and cardiovascular and cerebrovascular disease prevention properties^7–9^. Moreover, anthocyanin plays vital roles in attracting pollinators and seed dispersers, as well as protecting plants from abiotic and biotic stresses^8,10–13^. The anthocyanin biosynthesis pathway and the molecular mechanisms of anthocyanin accumulation are conserved and complicated in plants^5,14^. Briefly, it initiates with the synthesis of naringenin chalcone mediated by chalcone synthase (CHS), using 4-coumaroyl-CoA and malonyl-CoA as substrates. Subsequently, chalcone isomerase (CHI) converts naringenin chalcone to naringenin. Naringenin is then converted into dihydrokaempferol by flavanone 3-hydroxylase (F3H), which can be further hydroxylated into dihydroquercetin or dihydromyricetin by either flavonoid 3’-hydroxylase (F3’H) or flavonoid 3’,5’-hydroxylase (F3’5’H), respectively. Dihydroflavonol 4-reductase (DFR) converts the three dihydroflavonols into colorless leucoanthocyanidins, which are then transformed into colored anthocyanidins by anthocyanidin synthase (ANS). Finally, members of the glycosyltransferase enzyme family, such as flavonoid 3-O-glucosyltransferase (UFGT), attach sugar molecules to anthocyanidins, and the anthocyanidins may undergo further acylation by acyltransferases with aromatic acyl groups.

Nowadays, some candidate genes related to anthocyanin biosynthesis have been identified^7,13,15–19^. For example, Hayashi *et al*.^9^ have crossed a doubled haploid line of the turnip *Brassica rapa* cv. ‘Iyo-hikabu’, which is pigmented with anthocyanin, with a Chinese cabbage inbred line, ‘Y54’, which lacks anthocyanin pigmentation, and identified a novel locus (*Anp*) on chromosome A07^9^ related with anthocyanin synthesis based on a bulked segregant analysis. Burdzinski and Wendell (2007) identified three markers linked to anthocyaninless, forming a linkage group spanning 46.9 cM, which were assigned to *Brassica rapa* linkage group R09^20^ based on 177 F2 offspring. Additionally, the *pur* gene, responsible for regulating purple leaf color, was successfully mapped to the end of chromosome A03 using an F2 population^21^. An insertion and deletion (InDel) marker developed from deletion/insertion in the promoter region of bHLH49 in the F2 population was found to significantly correlate with the stalk color trait^2^. *EGL3*, a positive regulator gene with potentially epistatic function, was localized to mediate the anthocyanin biosynthesis^1^. Two candidate genes controlling anthocyanin accumulation were identified in the F2 population derived from a cross between Zicaitai and caixin^5^, and they were homologous with *AtEGL3*, *BrEGL3.1* and *BrEGL3.2* genes. Although several studies have characterized anthocyanin in *Brassica* crops, there is limited information on the genes involved in anthocyanin biosynthesis in Zicaitai.

Understanding the genome structure and identifying candidate genes related to anthocyanin biosynthesis is crucial for Zicaitai. However, the lack of a high-quality reference genome for Zicaitai makes it challenging to identify candidate genes associated with important agronomic traits and breed excellent Zicaitai varieties. Hence, we have generated a chromosome-level genome assembly of Zicaitai using PacBio long-reads, Illumina short reads, and Hi-C sequencing data first in this study. The assembled genome has a total length of 474.12 Mb, with a scaffold N50 length of 43.82 Mb, and 90.88% of the genome sequence was successfully anchored onto ten pseudochromosomes. Through a combination of *ab initio* gene predictions, RNA-seq, and homologous protein evidence, a total of 42,051 protein-coding genes were identified, and 41,942 of them were functionally annotated. The genome sequence provides a valuable resource for exploring the molecular basis of agronomic traits in Zicaitai and will further facilitate its genetic improvements.

## Methods

### Sample collection

Young fresh leaves of Zicaitai were collected from one sample individual grown in a greenhouse in Guangzhou, Guangdong, China (N 23°06’, E 113°15’), and immediately frozen in liquid nitrogen for genomic DNA and RNA extraction.

### DNA extraction and sequencing

Total high molecular weight (HMW) genomic DNA was extracted from Zicaitai young fresh leaves using the Tiangen Extraction Kit (Tiangen Biotech (Beijing) Co., Ltd.) for whole genome sequencing. The extraction process was according to the cetyltrimethylammonium bromide (CTAB) method, and concentration was ascertained by the Quant-iT PicoGreen® assay (Invitrogen, Waltham, MA, USA). The quality and quantity of the DNA samples were assessed using an ultraviolet spectrophotometer at 260 nm and 280 nm wave lengths. The DNA was fragmented with a Covaris M220 Focused-ultrasonicator Instrument. For genomic DNA sequencing, we employed three different approaches at Novogene Co., Ltd., Beijing, China. Firstly, DNA PCR-free libraries with insert sizes of 350 bp were constructed using the NEBNext Ultra DNA library Pre-Kit for Illumina short-reads sequencing. The resulting barcoded library were sequenced on the Illumina Hiseq 4000 platform to generate paired-end 150-bp reads. Subsequently, all the obtained reads were quality controlled by trimming adaptor sequences and low-quality reads using NGSQC v2.3^22^ (-q 20, https://github.com/mjain-lab/NGSQCToolkit). Secondly, single-molecule real-time (SMRT) PacBio libraries were constructed using the PacBio 15-kb protocol and sequenced with a Pacbio Sequel IIe platform. Lastly, the Hi-C library was generated using the restriction endonuclease DpnII. The DpnII-digested chromatin was labeled with biotin-14-dATP, and *in situ* DNA ligation was performed. The DNA underwent extraction, purification, and shearing. After A-tailing, pull-down, and adapter ligation, the DNA library was sequenced on the Illumina Hiseq 4000 platform. The total data generated from long-read sequencing was 35.29 Gb, and the total data generated from short-read sequencing was 10.06 Gb (Table 1).

**Table 1:** Statistics of sequencing data generated for the Zicaitai genome assembly.

Libraries	Insert size (bp)	Total data (G)	Read length (bp)	Sequence coverage (X)
Illumina reads	350	10.06	150	18.12
PacBio reads	—	35.29	—	63.55
Total	—	45.35	—	81.67

### RNA extraction and sequencing

Simultaneously, fresh root, stem, leaf, flower, and pod of the same Zicaitai individual were collected for transcriptome sequencing. Total RNA was extracted using the TRIzol reagent (Thermo Fisher Scientific, MA, USA) according to the manufacturer’s protocol. RNA-seq library were sequenced on an Illumina Novaseq 6000 platform with paired-end 150 bp reads. The adapters and low-quality reads of the raw sequence reads were trimmed using NGSQC v2.3^22^ (-q 20, https://github.com/mjain-lab/NGSQCToolkit). A total of ~15 Gb raw reads were yielded and used for the gene prediction.

### Genome size estimation and assembly

For quality control, the adapter sequences and low-quality reads obtained from Illumina Hiseq 4000 platform were filtered using NGSQC v2.3^22^ (-q 20, https://github.com/mjain-lab/NGSQCToolkit) and Trimmomatic v0.4^23^ (adapter:2:30:10:2:True LEADING:3 TRAILING:3 MINLEN:50). The genome size was then estimated by using GenomeScope v2.0^24^ and the 17-mer analysis with Jellyfish^25^ v2.2.7 (-C -m 17 -s 100 m) based on all the remaining reads. The final genome size was estimated to be 555.32 Mb.

**Fig. 2:**
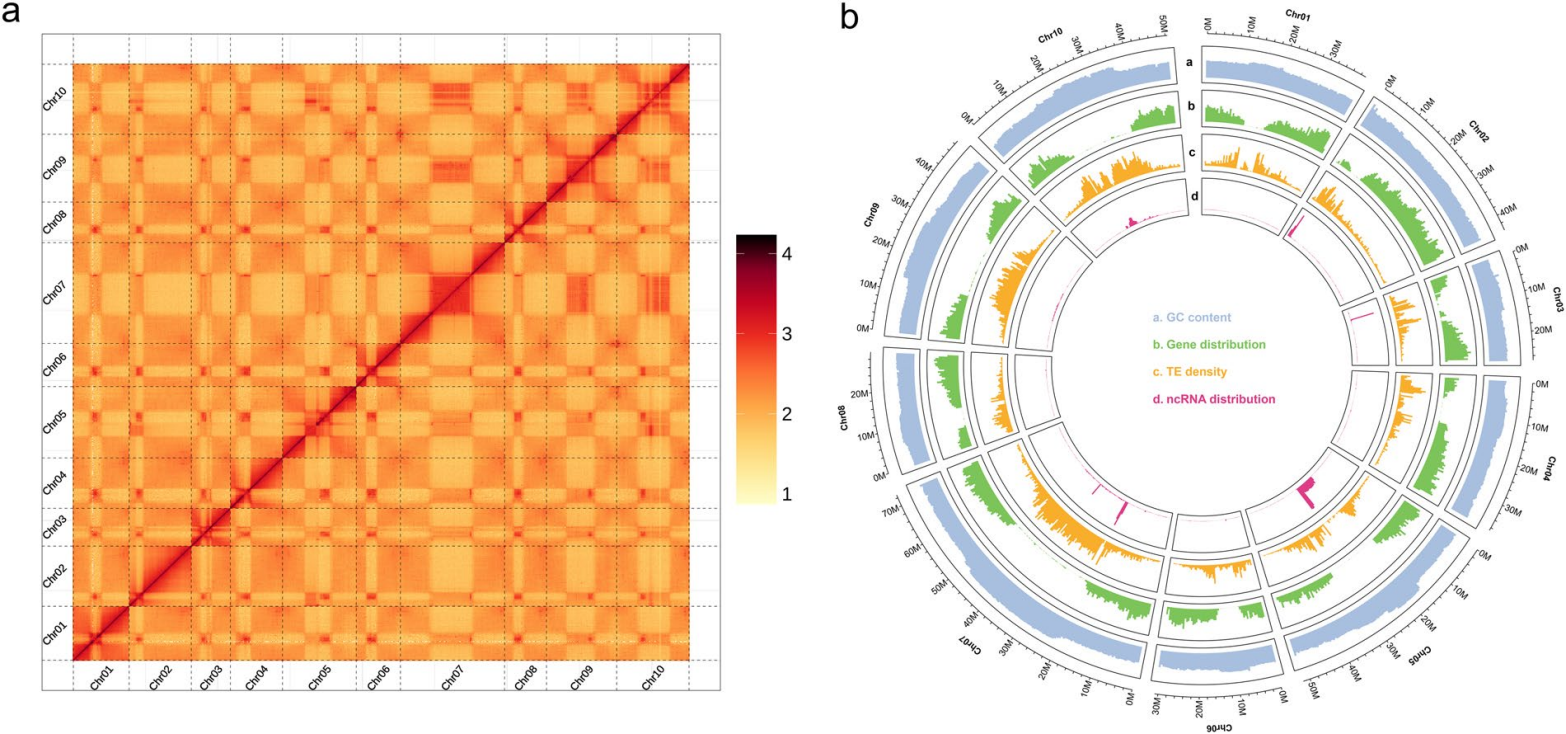
Hi-C chromatin interaction map and circos plot of the genome assembly. (**a**) Hi-C chromatin interaction map of the Zicaitai assembly. (**b**) The circos plot of genome characteristic of the Zicaitai. The rings from outside to inside indicate are: (a) GC content, (b) gene distribution, (c) TE density, and (d) ncRNA distribution in ten different chromosomes.

For the genome assembly of Zicaitai, Hifiasm v0.16.1^26^ was first used to assemble the initial assembly with PacBio CCS sequences. Secondly, NextPolish v1.3.1^27^ was then applied three times to polish the draft genome assembly using Illumina short reads. Then, ALLHiC^28^, a Hi-C scaffolding pipeline, was used to align Hi-C reads to the draft assembly and subject them to quality control. Finally, 3D-DNA v180419^29^ was used to anchor primary contigs into chromosomes, and ambiguous fragments were removed manually with Juicebox v1.13^30^, a visualization software for Hi-C data. All of the above software runs with default parameters. The final genome assembly of Zicaitai was 474.12 Mb with a scaffold N50 of 43.82 Mb. The Hi-C analyses scaffolded ten pseudo-chromosomes (Fig. 2a), anchoring 90.88% (430.88 Mb) of the genome assembly. The average GC content of Zicaitai genome assembly was 38.54% (Table 2, Fig. 2b).

**Table 2:** Summary statistics of the Zicaitai genome assembly.

Genome assembly			
Primal assembly		Final assembly	
Genome assembly size	493.78 Mb	Genome assembly size	474.12 Mb
Number of contigs	608	Number of contigs	604
Number of scaffolds	608	Number of scaffolds	576
Contig N50	21.56 Mb	Contig N50	21.56 Mb
Scaffold N50	21.56 Mb	Scaffold N50	43.82 Mb
BUSCO completeness	99.30%	BUSCO completeness	—
Number of chromosomes	—	Number of chromosomes	10
GC content (%)	38.54	Sequences assigned to pseudo-chromosomes (%)	90.88 (430.88 Mb)

### Genome completeness

The genome completeness was evaluated with BUSCO v5.4.7^31^, searching against the embryophyta_odb10 database. The analysis found 99.30% (single-copied genes: 84.70%, duplicated genes: 14.60%), 0.20%, and 0.50% of the 42,051 projected genes in this genome as complete, fragmented, and missing sequences, respectively, indicating a highly complete genome assembly. Moreover, 242 genes were assembled in the CEGMA^32^ database (248 cor genes), suggesting a completeness score of 97.58%.

### Genome annotation

**Fig. 3:**
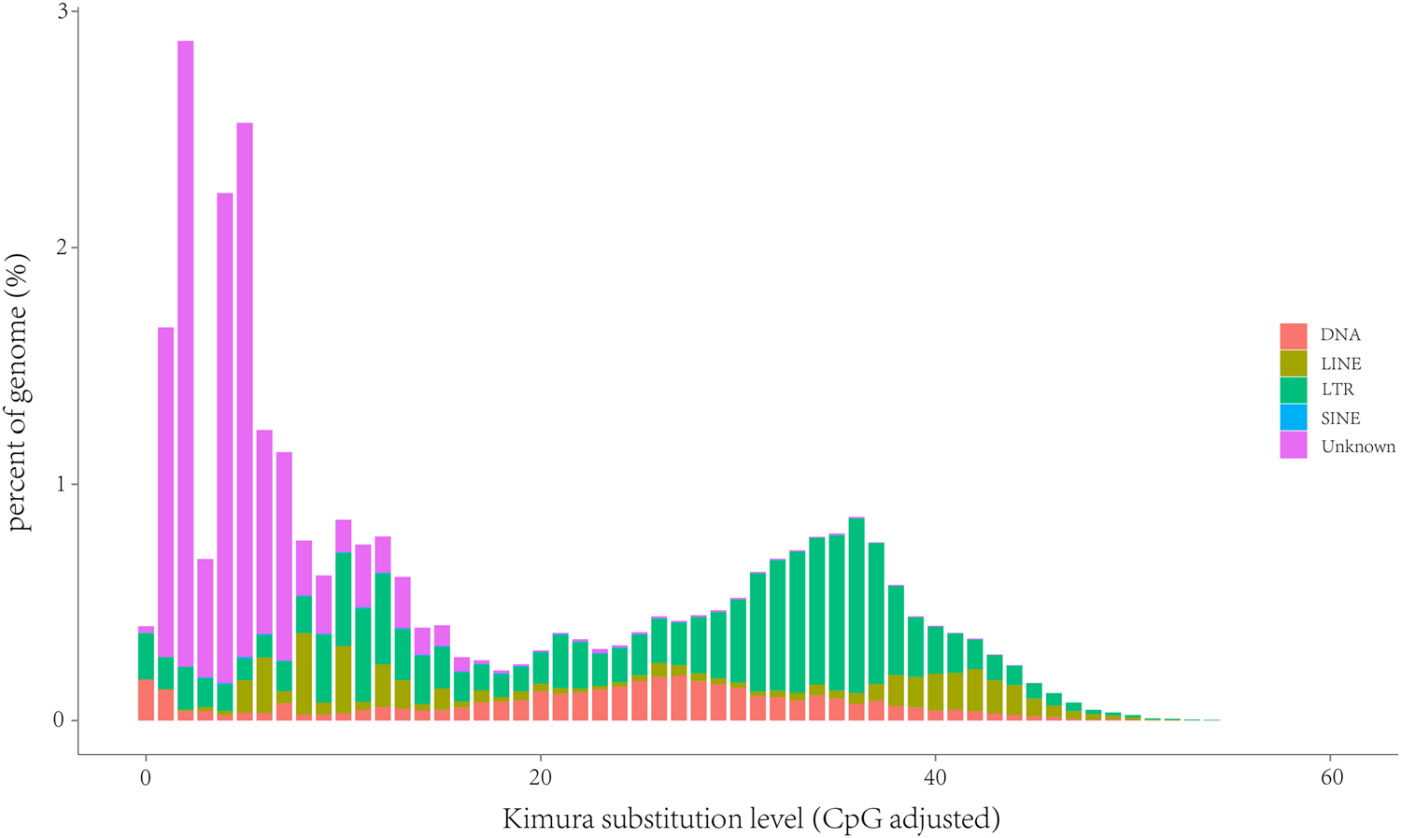
Repeat landscape plots illustrating TE accumulation history for Zicaitai genome, based on Kimura distance-based copy divergence analyses, with sequence divergence (CpG adjusted Kimura substitution level) illustrated on the x-axis, percentage of the genome represented by each TE type on the y-axis, and transposon type indicated by the colour chart on the right-hand side. CpG, region of DNA where a cytosine nucleotide is followed by a guanine nucleotide; LINE, long interspersed nuclear element; LTR, long terminal repeat; SINE, short interspersed nuclear element.

The repetitive elements, protein-coding genes, and non-coding RNAs (ncRNAs) of the Zicaitai genome was annotated. The whole genome repeats were identified using a combined strategy based on homology alignment and *de novo* search. Tandem Repeat was extracted using TRF^33^ by *ab initio* prediction. The homolog prediction commonly used Repbase^34^ database employing RepeatMasker and RepeatProteinMask with default parameters to extract repeat regions. *Ab initio* prediction built the *de novo* repetitive elements database by LTR_FINDER^35^, RepeatScout^36^, and RepeatModeler2^37^ (-LTRStruct), and then all repeat sequences with lengths more than 100 bp and gap ‘N’ less than 5% constituted the raw transposable element (TE) library. A total of 288.72 Mb repetitive elements were identified, constituting 60.89% of the total genome sequence. The most abundant repeating element was long terminal repeats (LTR, 47.96%), and unknown repeats (43.71%), followed by DNA transposons (6.08%) (Fig. 2b, Table 3, Fig. 3).

**Table 3:** Statistics and classification of repetitive elements in the Zicaitai genome.

Statistics of repetitive elements		
Type	Repeat Size (Mb)	Percentage of genome (%)
TRF	93.96	19.82
Repeatmasker	280.53	59.17
Proteinmask	42.40	8.94
Total	288.72	60.89
**Classification of repetitive elements**		
**Type**	**Length (Mb)**	**Percentage of genome (%)**
DNA	28.83	6.08
LINE	10.14	2.14
SINE	0.50	0.11
LTR	227.38	47.96
Unknown	24.70	43.71

**Fig. 4:**
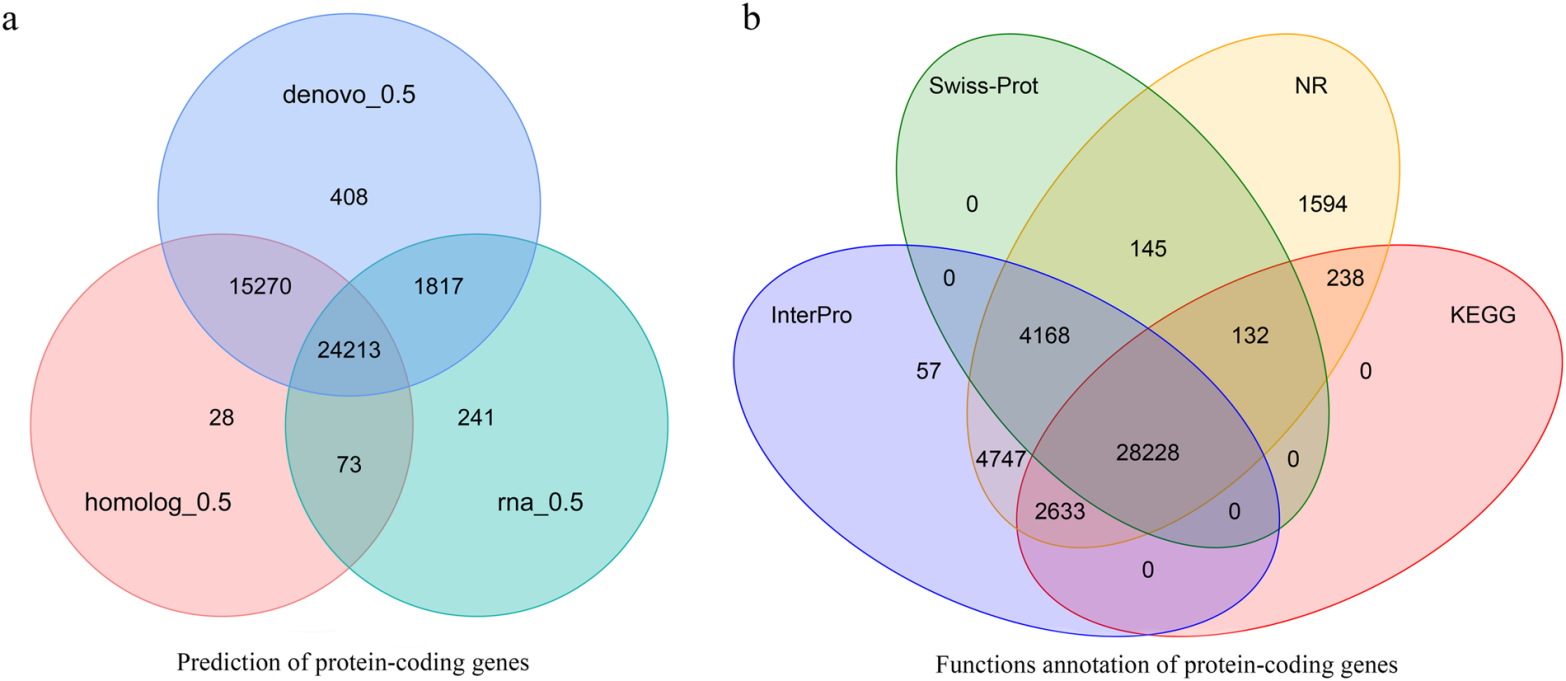
Prediction and annotation of protein-coding genes in the Zicaitai genome.

*De novo* gene prediction, homology-based prediction, and RNA-seq were applied for the annotation of protein-coding genes. The repeat-masked genome was analyzed using Augustus v2.4^38^, GlimmerHMM v3.0.4^39^, Geneid v1.4.5^40^ and Genscan^41^ for *de novo* gene prediction. The protein sequences of *Cruciferae* species were downloaded from the NCBI Database as references for homology-based prediction. Transcriptome assemblies were also generated with Trinity v2.5.1^42^ for the genome annotation. A consensus gene set was created by integrating the genes predicted by the aforementioned three methods using EVidenceModeler v1.1.1^43^. Finally, a total of 42,051 protein-coding genes were predicted for the Zicaitai genome by combining the evidence from the transcriptome, *ab initio*, and homology-based predictions (Fig. 4a). The average length of the predicted protein-coding gene was 2,001 bp. The average lengths of the exon and intron were 228 bp and 235 bp, respectively. Each gene has an average of 4.82 exons. (Table 4). Gene functions were assigned according to the best match by aligning the protein sequences to the Swiss-Prot, GO, NR, InterPro, Pfam, and KEGG databases, respectively. A total of 41,942 (99.74%) genes were functionally annotated (Table 4, Fig. 4b).

**Table 4:** Prediction and annotation of protein-coding genes in the Zicaitai genome.

Protein-coding genes		Number of genes with annotation	
Total gene numbers	42,051	NR database	41,885
BUSCO completeness	99.30%	Swiss-Prot database	32,673
Average transcript length (bp)	2,001	KEGG database	31,231
Average CDS length (bp)	1,101	InterPro database	39,833
Average exons per gene	4.82	Pfam database	30,755
Average exon length (bp)	228	GO database	23,829
Average intron length (bp)	235	Swiss-Prot database	32,673

For tRNA prediction, the program tRNAscan-SE^44^ was used, while for rRNA prediction, Blast^45^ program was used with relative species’ rRNA sequences as references. Other ncRNAs, including miRNAs and snRNAs, were identified by searching against the Rfam^46^ database using the infernal v1.1^47^ software with default parameters. Finally, a total of 1,894 non-coding RNAs were predicted, including 4,885 transfer RNAs (tRNAs), 6,402 ribosomal RNAs (rRNAs), 511 micro-RNAs (miRNAs), and 2,774 small nuclear RNAs (snRNAs) (Table 5, Fig. 2b).

**Table 5:** Statistics and classification of non-coding RNAs identified in the Zicaitai genome.

Type		Copy number	Average length (bp)	Total length (kb)	Percentage of genome (%)
miRNA		511	123.66	63.19	0.01
tRNA		4,885	75.66	369.60	0.08
rRNA	rRNA	32,201	355.12	11,435.22	2.41
	18S	4,417	1,533.10	6,771.71	1.43
	28S	14,742	138.06	2,035.33	0.43
	5.8S	3,794	395.66	1,501.14	0.32
	5S	9,248	121.89	1,127.04	0.24
snRNA	snRNA	1,387	111.68	154,901	0.03
	CD-box	1,095	104.64	114,585	0.02

## Data Records

The Illumina short reads, PacBio long-reads, Hi-C sequencing data, and RNA-seq data used for the genome assembly and annotation have been deposited in the NCBI Sequence Read Archive (SRA) database with the accession number SRP441633^48^. The chromosomal-level genome assembly sequence and annotation information have been deposited in the Figshare database^49^. The chromosomal-level genome assembly sequence was deposited in the GenBank database of NCBI with accession number JAUJLN000000000^50^.

## Technical Validation

### Evaluating the quality of the genome assembly

We evaluated the quality and completeness of the Zicaitai genome assembly using two approaches. First, we mapped short-reads to the genome to verify the accuracy, yielding mapping rates of 99.22%, which suggests the accuracy of the Zicaitai genome assembly. Second, BUSCO analysis found 99.30% of the 1,614 single-copy orthologues in the embryophyta_odb10 database to be complete (84.70% single-copied genes and 14.60% duplicated genes), with 0.2% fragmented and 0.5% missing (Table 4), indicating a remarkably complete assembly of the Zicaitai genome. Additionally, the whole-genome high long terminal repeat (LTR) assembly index (LAI) score is an important indicator of genome assembly quality and completeness. In this study, the LAI score for Zicaitai genome assembly was 10.14, indicating that the assembly quality of Zicaitai reached the reference genome level.

### Gene annotation validation

**Fig. 5:**
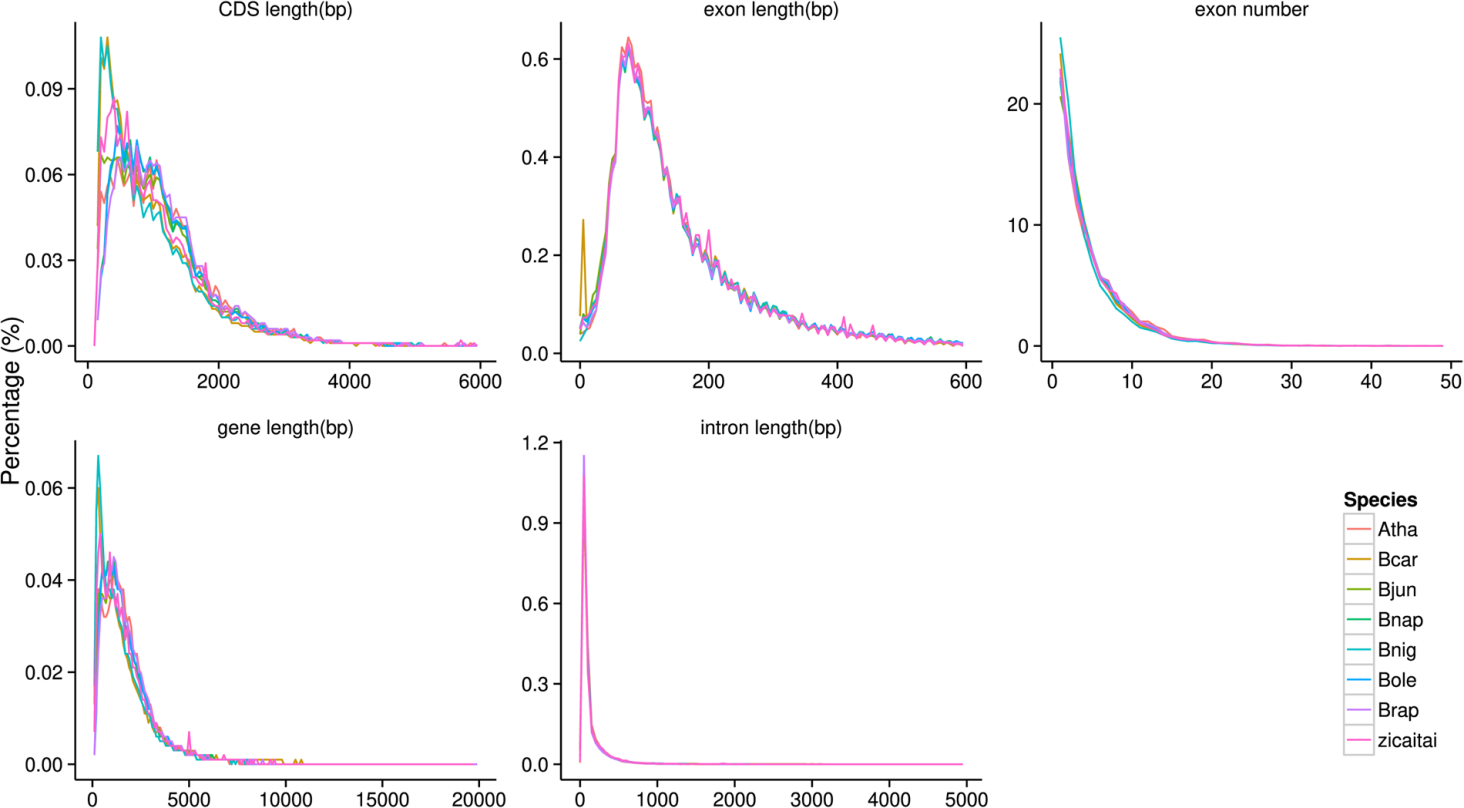
Genetic components of the Zicaitai genome and seven other *Cruciferae* species.

To validate gene annotation, we studied the structure and number of genes in Zicaitai and seven other *Cruciferae* species based on protein annotation sequences retrieved from NCBI, including *Brassica rapa* (Brap), *Brassica napus* (Bnap), *Brassica juncea* (Bjun), *Brassica nigra* (Bnig), *Brassica oleracea* (Bole), *Brassica carinata* (Bcar), and *Arabidopsis thaliana* (Atha). A total of 42,051, 100,829, 96,553, 41,049, 57,386, 97,148, 43,923, and 27,221 protein-coding genes were identified in Zicaitai, Bjun, Bnap, Brap, Bnig, Bcar, Bole, and Atha, respectively (Table 6, Fig. 5). Except for tetraploid plants (Bnap, Bjun, and Bcar), the gene numbers of other diploid species were similar (about 40,000). The average lengths of transcripts, CDS, exons, and introns in Zicaitai and the other seven *Cruciferae* species were found to be almost identical. Additionally, the average number of exons per gene was also found to be equivalent across all species.

**Table 6:** Gene component analysis of the genomes of Zicaitai and seven other *Cruciferae* species, namely *Brassica rapa* (Brap), *Brassica napus* (Bnap), *Brassica juncea* (Bjun), *Brassica nigra* (Bnig), *Brassica oleracea* (Bole), *Brassica carinata* (Bcar), and *Arabidopsis thaliana* (Atha).

Species	Gene number	Average transcript length(bp)	Average CDS length(bp)	Average exons per gene	Average exon length(bp)	Average intron length(bp)
Zicaitai	42,051	2,001.16	1,101.46	4.82	228.29	235.23
Bjun	100,829	2,023.83	1,161.33	4.84	239.88	224.54
Bnap	96,553	2,021.74	1,201.60	4.86	247.46	212.70
Brap	41,049	2,042.79	1,260.81	5.03	250.79	194.16
Bnig	57,386	1,734.89	1,042.26	4.42	236.02	202.76
Bcar	97,148	2,374.57	1,004.92	4.60	218.29	380.08
Bole	43,923	2,033.70	1,195.25	4.87	245.40	216.62
Atha	27,221	1,902.49	1,232.41	5.17	238.26	160.60

**Fig. 6:**
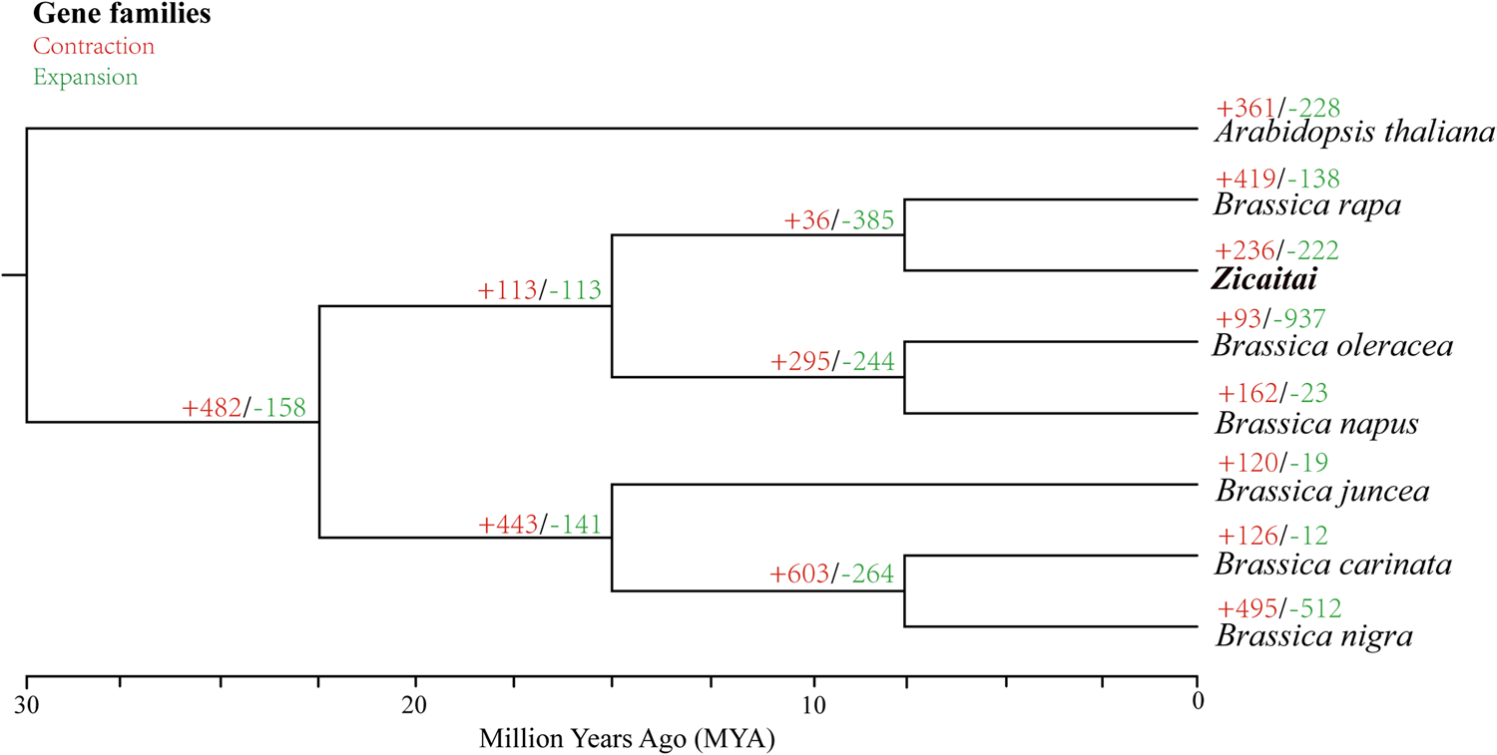
Phylogenetic tree and gene families expansion and contraction of Zicaitai and seven other *Cruciferae* species. The scale at the bottom of the figure represents the divergence time.

**Fig. 7:**
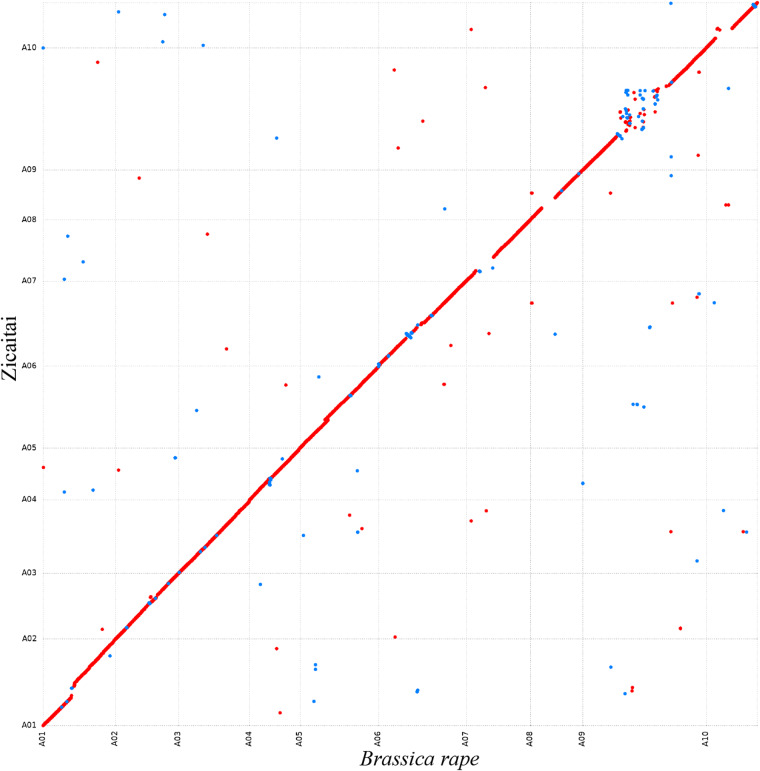
Dot plot represents an alignment of two different genomes of *Brassica rapa* (x-axis) and Zicaitai (y-axis). Forward matches are shown in red, while reverse matches are shown in blue.

OrthoFinder v2.5.4^51^ was used to infer sequence orthology. Phylogenetic trees were constructed using single copy gene family, and the differentiation time was estimated using the r8s program^52^. Based on the time tree, expansions and contractions of the gene family of Zicaitai and seven other Cruciferae species was estimated using CAFE v5^53^ with a *p* value of 0.01. Finally, 236 and 222 gene families experienced expansions and contractions were found in Zicaitai, respectively (Fig. 6). Moreover, we have compared the genome sequences of Zicaitai and *Brassica rape* (Brara_Chiifu_V4.0), and the results indicated a significant degree of collinearity for the two genome sequences of Zicaitai and *Brassica rape*, with the exception of certain contigs located on chromosome 9 (Fig. 7).

## Data Availability

This work did not utilize a custom script. Data processing was carried out using the protocols and manuals of the relevant bioinformatics software.
